# Trajectories of a set of ten functional somatic symptoms from adolescence to middle age

**DOI:** 10.1186/s13690-017-0178-8

**Published:** 2017-03-06

**Authors:** Tapio Nummi, Pekka Virtanen, Päivi Leino-Arjas, Anne Hammarström

**Affiliations:** 1 Faculty of Natural Sciences, University of Tampere, 33014 Tampere, Finland; 2 School of Health Sciences, University of Tampere, 33014 Tampere, Finland; 3 Finnish Institute of Occupational Health, Centre of Expertise for Health and Work Ability, Helsinki, Finland; 4 Department of Public Health and Caring Sciences, Uppsala University, Uppsala, Sweden

**Keywords:** Finite mixtures, Logistic regression, Longitudinal data, Multivariate trajectory analysis, Pain

## Abstract

**Background:**

Functional somatic symptoms (FSS), or symptoms without a clear medical explanation are a considerable challenge for health care systems. There is no general consensus as to which symptoms should be regarded functional. Few longitudinal studies on the development of FSS exist and these have mainly been based on the assumption that the factorial structure of a FSS scores variable remains invariant over time. When the analysis covers longer periods of the life course, this may be challenged. The aim of our study was to investigate how ten functional somatic symptoms (FSS) evolve when individuals are ageing.

**Methods:**

The data of the Northern Swedish Cohort (*n* = 1001) from questionnaire surveys at ages 16, 18, 21, 30 and 42, were analysed. Participation rates remained very high over the five surveys. The list of symptoms included backache, breathlessness, dizziness, fatigue, headache or migraine, nausea, overstrain, palpitations, sleeplessness and stomach ache. We used multivariate trajectory analysis (TA) with logistic broken-stick regression models to describe sub-groups in the data. In multivariate TA the joint development of the set of item variables can be investigated. There is no need to construct a special FSS summary score variable.

**Results:**

Four well separated trajectories were identified. In two groups, healing symptoms (25.4% of the sample) and low symptom load (32.2% of the sample), the symptom level stayed relatively low in adulthood. In the third group of high symptom load (17.2%) the probability of having symptoms was high for all FSS variables. In the fourth group of increasing symptoms (25.3%) the level of symptoms was first intermediate, but increased markedly with age.

**Conclusions:**

Instead of a single FSS score we were able to assign each individual to one of four trajectories described jointly by 10 separate symptoms. The profile of development, but not the probability level, was rather similar over the symptoms within the trajectories, with few exceptions. The results provide better understanding of the longitudinal development of the symptoms from the adolescence to the middle age.

## Background

Over decades, tens of questionnaires have been constructed to measure the number, the frequency and the severity of somatic symptoms in various client or population groups (see [18]). The questionnaires are principally intended to produce a single quantitative indicator of the latent variable that, depending on the theoretical assumptions, can be conceptualized in several ways. The review referred to above assumes the term ‘functional somatic’, implying some kind of physiological disturbance, but the symptoms have also been called ‘medically unexplained’ in terms of a lack of diagnosed disease, as ‘stress’ in terms of a strain in the individual-environment relationship, or as ‘psychosomatic’ or ‘internalized’ in terms of underlying mental health disorder. Our choice in the present paper is functional somatic symptoms (FSS). It is not clear from the literature which specific symptoms should be considered as FSS. Any one of the common symptoms presented by patients seeking care or reported in a questionnaire might be considered as arising from a disease of a particular organ system. However, the more symptoms a person has, the less likely it apparently is that these reflect the presence of a somatic disease [9].

In the research context, FSS summary score indices have mostly been analysed with statistical methods intended for cross-sectional data such as logistic regression, principal component analysis, factor analysis and analysis of correlation coefficients (e.g. [6, 8, 16]). There are only few studies that analyse FSS in longitudinal settings. Rousseau et al. [14] and Janssens et al. [3] have used latent growth mixture model [11] when investigating the trajectories of FSS and their association with background characteristics in adolescence. The trajectory analysis by Nagin [12] was used by Mulvaney et al. [10] in the study of children suffering from abdominal pain. Usually these trajectory analyses found 3 to 4 groups sizes varying depending on the statistical models used, the score variable utilized and the age period investigated.

Longitudinal analysis of FSS scores is based on the assumption that the factorial structure of the score variable remains invariant over time. FSS score has been analysed, for example, in a study of the Northern Swedish Cohort [2]. This cohort is also the source of the present study. In order to expand our understanding of FSS, we decided to test a different approach, which takes into account the fact that individual symptoms can develop in different ways at different stages of life. Thus, our aim was to investigate how ten symptoms assumed as functional evolve when individuals are ageing from age 16 to age 42 by applying multivariate trajectory analysis of the symptom set.

## Methods

The Northern Swedish Cohort consists of pupils who studied their last year of compulsory school in a medium-sized Swedish industrial town in 1981. Data were collected at ages 16, 18, 21, 30 and 42 with surveys comprising their health and health behaviour, labour market experiences, family situation and socioeconomic conditions. The baseline survey at age 16 took place in classrooms and the follow-up surveys were conducted in classmate reunions or by post or telephone. At the latest follow-up survey in 2007 the participation rate was still 94.3% (*n* = 1010; 522 men and 488 women). The study was approved by the Regional Ethical Review Board in Umeå, Sweden.

There were few dropouts or missing data and the effective sample size for this study was *n* = 1001 (519 or 52% men and 482 or 48% women). The set of variables for measuring FSS was constructed by a panel consisting of 25 health professionals (for more details see [2]). For each of the listed 42 candidate symptoms, the panel was asked to judge whether the symptom belonged to FSS or not. The following ten symptoms received the highest support: 1) stomach ache other than heartburn, gastritis or gastric ulcer (96%); 2) headache or migraine (80%); 3) fatigue (76%); 4) dizziness (72%); 5) palpitations (72%); 6) nausea (68%); 7) sleeplessness (68%); 8) backache, hip pain or sciatica (64%); 9) breathlessness (64%) and 10) overstrain (64%). In the survey the occurrence of these 10 symptoms during the past 12 months was inquired using three response alternatives (no; yes, light; yes severe). For our analyses each symptom variable was dichotomized as no vs. yes.

The longitudinal data of FSS were analysed using trajectory analysis (TA), which can be used to model the unobserved heterogeneity in longitudinal data. The TA by Nagin [12, 13], Jones and Nagin [5] and Jones et al. [4] applies the generalized linear models theory (exponential family of distributions) with Finite Mixtures under the assumption that observations within a given trajectory are independent. Our main analysis used the multivariate version of this basic TA, where outcomes are related but distinct variables (see e.g. [4, 13]). The computations were carried out by R package Flexmix [7].

**Fig. 1:**
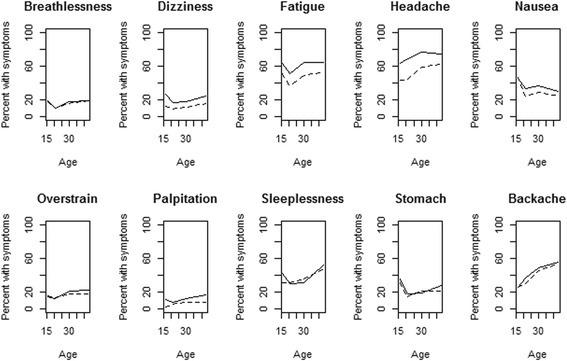
Plots of percentage of subjects reporting functional somatic symptoms as a function of age. Solid curve is for women and dotted curve is for men

As can be observed from Fig. 1, the overall trend is that the prevalence of symptoms first decreases and then increases with age. However, some symptoms increase only slightly (breathlessness, dizziness, overstrain and palpitation) and some even decrease (nausea). The highest percentages were obtained for fatigue and headache. It is further observed that age 21 is a kind of turning point, from which most FSS variables develop fairly stably over adulthood. The basic linear model that can be used for modelling such development is the so-called broken-stick model that joins two regression lines at a given knot point [15]. Here we take the knot point as 21 years of age. Figure 1 also shows that FSS are fairly similar for both genders, but for women the level tends to be higher.

This is in line with many earlier studies and must also be accounted for when constructing statistical models. The joint model used for each of the 10 symptoms variables takes the form of the broken-stick

$$ \mathrm{logit}\left(\mathrm{p}\right)=\mathrm{b}0 + \mathrm{b}1\ \mathrm{sex} + \mathrm{b}2\ \mathrm{t} + \mathrm{b}3\ \mathrm{t}+, $$

where t is age and t + is the positive part of (t −21) and zero otherwise. This formulation splits the model into two parts. The first covers the age period (16, 21) and the second part adds the age period [21, 42]. The multivariate trajectory analysis model consists of 10 logistic regression models introduced above, where two regression lines are joined together at the knot point t = 21 and the intercept term is estimated separately for men and women. Note that in this kind of multivariate formulation the full potential of all the 10 variables are used in TA. This is in contrast to more common FSS summary score analysis that can potentially mask some important development of a single item variables in the analysis, because the symptoms behave in different ways at different periods of age.

## Results

**Fig. 2:**
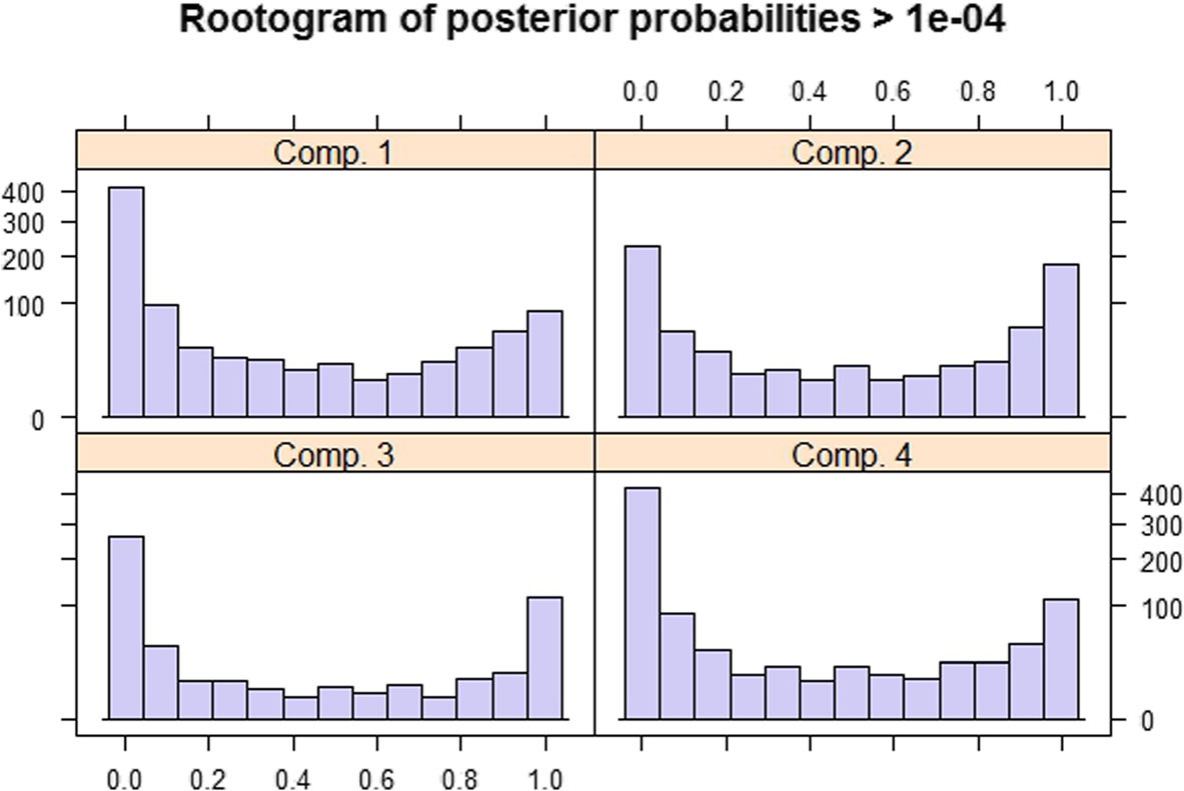
Rootogram plot of posterior probabilities

The number of trajectories to be tested was k = 1, …, 7. To ensure convergence, each run was repeated 10 times using different starting values. The obtained values for the information criteria BIC were then 41011.46, 38655.49, 38490.46, 38375.48, 38500.89, 38628.52 and 38799.41. The minimum BIC = 38375.48 was clearly attained when k = 4 and this is therefore taken as the number of trajectory groups for our analysis. The size of the trajectories varied between 17 and 32%; the estimated mixture proportions were p1 = 0.254, p2 = 0.322, p3 = 0.172 and p4 = 0.253. The so-called rootogram plot of posterior probabilities (Fig. 2) confirmed that the four groups were very well separated.

**Fig. 3:**
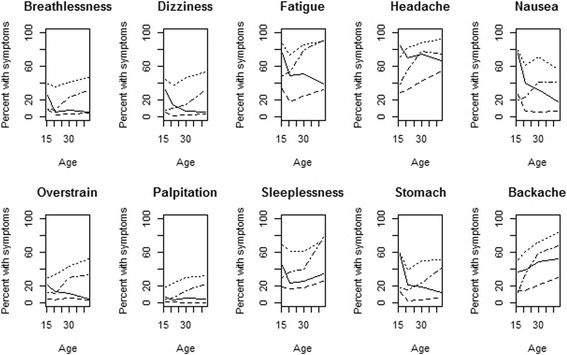
Plot of 10 FSS variables for each trajectory group as a function of age. Solid curve is for the group of healing symptoms,* dashed curve* for the group of permanently low symptom load, dotted curve for the group of permanently high symptom load and the *semi-dashed curve* is for the group of increasing symptoms

**Fig. 4:**
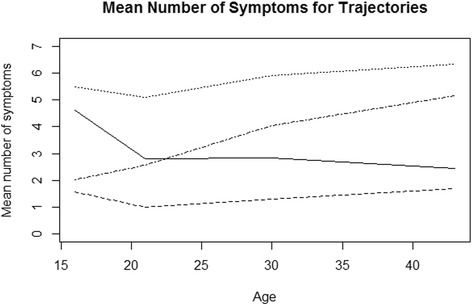
Mean number of symptoms by age. Solid curve is for the group of healing symptoms, dashed curve for the group of permanently low symptom load, dotted curve for the group of permanently high symptom load and the semi-dashed curve is for the group of increasing symptoms

Figure 3 illustrates the development of each symptom in each trajectory group, and estimates of the model parameters are displayed in Table 1. Using this information together with the mean of the number of symptoms as a function of age (Fig. 4), we characterised the trajectory groups as follows.

**Table 1:** Estimates of the parameters for the broken-stick model for each trajectory group

Group	Par.	Breath.	Dizzi.	Fatigue	Head.	Nausea	Overst.	Palpi.	Sleep.	Stomach	Back
Healing	b0	3.919*	3.405*	4.647*	3.850*	5.772*	0.373	−0.646	2.836*	5.452*	−1.119
	b1	0.194	−1.149*	−0.540*	−0.723*	0.395*	−0.184	−1.238*	−0.221	0.032	−0.062
	b2	−0.318*	−0.230*	−0.207*	−0.124*	−0.306*	−0.098	−0.095	−0.186*	−0.325*	0.036
	b3	0.322*	0.182*	0.191*	0.120*	0.258*	0.058	0.100	0.211*	0.299*	−0.010
Low	b0	2.340	1.958	2.094*	−0.792	3.276*	−2.897	−1.072	−0.397	4.236*	−1.981*
	b1	−0.381	−0.850*	−0.593*	−0.700*	0.237	−0.543	−1.243	−0.494*	−0.482	0.079
	b2	−0.279*	−0.265*	−0.154*	0.024	−0.276*	0.008	−0.145	−0.048	−0.363*	0.012
	b3	0.319*	0.293*	0.188*	0.014	0.271*	−0.006	0.117	0.074	0.410*	0.028
High	b0	−0.301	0.781	4.744*	−0.565	3.409*	−1.538	−2.214*	1.642	2.801*	−1.192
	b1	−0.040	−0.510*	−0.422	−1.583*	−0.774*	0.388*	−0.582*	0.474*	−0.226	−0.159
	b2	−0.011	−0.050	−0.165*	0.152*	−0.110*	0.030	0.065	−0.071	−0.144*	0.082
	b3	0.030	0.083	0.222*	−0.110	0.098	0.007	−0.045	0.101	0.164*	−0.030
Increase	b0	−2.670*	−2.472	−0.453	−2.970*	−1.767	−2.925*	−4.206*	−0.824	−0.739	−6.335*
	b1	−0.047	−0.785*	−0.712*	−1.059*	−0.705*	−0.322	−0.916*	0.068	−0.190	−0.234
	b2	0.027	0.026	0.047	0.189*	0.053	0.065	0.095	−0.001	−0.041	0.285*
	b3	0.038	0.043	0.052	−0.151*	−0.022	−0.014	−0.031	0.085	0.103	−0.226*

Estimates for the male gender (difference from females) are denoted by b1

Coefficient b2 is the regression coefficient for age period (16, 21) and b2 and b3 jointly are coefficient estimates for the age period [21, 42]

Statistically significant estimates (at 5% level of significance) are marked with an asterisk

### Healing symptoms, 25.4%

For most variables the development curve first decreased and after age 21 either further decreased or stayed at the low level (in the model b2, b3 and Fig. 3). Although the probability of most of the symptoms was rather high in adolescence it became relatively low at the end of the follow-up in middle-age (Figs. 3 and 4). There were two exceptions: changes in the probability of palpitations were small throughout (b1 and b2) and that of backache increased moderately over time (Fig. 3). The level of the symptoms (b1) was lower for men considering the variables dizziness, fatigue, headache and palpitation. However, for nausea the constant term (b1) was significantly higher for men. The odds of developing symptoms of nausea for males in this group were about OR = 1.5 when compared to females.

### Low symptom load, 32.2%

The mean number of symptoms was lowest in comparison to other trajectories (Figs. 3 and 4), but after age 21 it tended to increase slightly (b2, b3, Figs. 3 and 4). The level of the symptoms was (significantly) lower in men than in women considering Dizziness, Fatigue, Headache and Sleeplessness (b1).

### High symptom load, 17.2%

In this group the level of the symptoms was high for each FSS variable, especially for fatigue, headache, nausea and sleeplessness (Fig. 3). For headache the curve increased slightly over the whole follow-up period (b2) and for fatigue and stomach ache (b2 and b3) it increased after the age 21. The level of the symptoms (b1) in men was (significantly) lower for dizziness, headache and nausea, but higher for overstrain (OR = 1.5) and sleeplessness (OR = 1.6) than in women.

### Increasing symptoms, 25.3%

The baseline level of the number of symptoms was low or intermediate, but increased by age especially for fatigue (Fig. 3), sleeplessness (Fig. 3), backache (Fig. 3, b2 and b3) and headache (Fig. 3, b2 and b3). The symptom level in men was significantly lower than in women for dizziness, fatigue, headache, nausea and palpitations (b1).

The groups of healing and increasing symptoms are slightly dominated by women (52.0 and 52.2%, respectively), while the group of low symptom load is dominated by men (57.6%). In the group of high symptom load the proportions are about the same as in the original sample.

In the group of increasing symptoms, the average number of symptoms clearly increased over time, but in the other groups, the number of symptoms remained fairly stable in adulthood. The highest overall number of symptoms by the end of the follow-up was obtained in the group of permanently high symptom load, and the second highest in the group of increasing symptoms. These two groups are interesting from the research point of view, but the deeper analysis goes beyond the scope of this paper.

## Discussion

We explored the trajectories of ten FSS over a 26-year time span among, originally, 16-year-old Swedes. Four distinct sub-groups were found, labelled as healing, low symptom load, high symptom load, and increasing symptoms. When summarizing the main findings we note that in the first two trajectory groups (healing and low symptom load), the symptom levels stay relatively low in adulthood. This applies for all symptoms. Slightly more than one half of the sample belonged to these groups with a favourable long-term development. Potentially more clinically relevant are the third group that comprises individuals that have a high level of FSS already in their teens, and the fourth group where the level of the symptoms is low or intermediate at baseline, but undergoes a steep increase till midlife. The latter two groups call for further research, because high levels of FSS may be associated with psychiatric disorders such as anxiety and depression, lower quality of life, and social problems [1, 10, 17] in addition to being themselves disturbing. However, a deeper analysis goes beyond the scope of this more methodologically oriented paper.

Our analysis revealed rather similar patterns for the various single symptom variables within the groups, but in the group of increasing symptom the increase is mainly due to increase of pain symptoms (Headache and Backache, b2 and b3) as well as fatigue and sleeplessness (Fig. 3). This suggests a somewhat different aetiology in symptoms formation.

Comparing our results to the few other trajectory analyses of FSS is difficult, since they are carried out among participants with a shorter follow-up in the youth and a single FSS score variable. Janssens et al. [3] studied the development of a score of seven FSS over a 7-years time span among Dutch teens aged from 11 to 16 years on average in the three waves of data collection. In their four-trajectory solution the group with low symptoms was the largest and the group with persistent FSS the smallest, similar to our findings. The group sizes were different, however, presumably due to the differences in age and length of follow-up between studies. The group with healing symptoms comprised about a quarter of the subjects in our study, while one tenth of the Dutch teens belonged to the group with decreasing symptoms. In the trajectory analysis by Mulvaney et al. [10] the long-term risk group consist of 14% of paediatric patients.

Longitudinal analysis of FSS is often based on the assumption that factorial structure of the score variable remains invariant over time. This was also tested in a study of the Northern Swedish Cohort [2] partly for the same variables that is considered in this study. However, in order to expand our understanding about the development of FSS we decided to test a different approach, which takes into account that individual symptoms can develop in different ways at different stages of life. So, the focus was not on the score variable; instead the aim is to learn to understand potential variation in the role of individual symptoms when the development from adolescence to middle age is elucidated.

As discussed in Beck [1], longitudinal studies must replace cross-sectional designs that use large and varied age ranges with studies that give us tools to understand the extent to which FSS are normative at certain development periods. Our results are also supported by the view of Wessey et al. (1999), who emphasize a dimensional classification of symptoms. This study uses good-quality longitudinal data. The sample size is adequate and the attrition rate is extremely low. In addition, the time period investigated is long enough to assess the development of symptoms from adolescence to adulthood. Here we have used the multivariate trajectory analysis model for FSS that, to our knowledge, has not been utilized earlier. The model gives us means to assess how the individual symptoms develop as a function of age for each identified trajectory group. The trajectories with their qualitative interpretation yield novel perspectives to the longitudinal development of the symptoms. The important limitation of the analysis method is that observations within each trajectory class are assumed to be independent within and between each variable. This may produce some small bias in parameter estimates of the trajectory model parameters. However, this small bias effect may not have a remarkable influence on the main conclusions of the study.

## Conclusions

It has been shown earlier that the factorial invariance over time applies to the FSS score variable that is based on seven FSS symptoms [2]. However, the full multivariate trajectory analysis of ten FSS variables applied here has the advantage that it takes into account the fact that individual symptoms within latent groups may not evolve on the same way for each study participant in each period of age. Instead of the single value of an FSS score variable we can assign each individual to one of the four trajectories, which gives a nice qualitative interpretation. This yields a better understanding about the longitudinal development of the symptoms and especially better tools for the identification of the risk group of individuals whose symptoms formation should be investigated in more detail in further studies.

## Abbreviations

FSS: Functional somatic symptoms
TA: Trajectory analysis
